# Genomic deletions on 16p11.2 associated with severe obesity in Brazil

**DOI:** 10.3389/fendo.2024.1495534

**Published:** 2025-01-17

**Authors:** Izadora Sthephanie da Silva Assis, Kaio Cezar Rodrigues Salum, Rafaela de Freitas Martins Felício, Lohanna Palhinha, Gabriella de Medeiros Abreu, Tamara Silva, Fernanda Cristina Carvalho Mattos, Eliane Lopes Rosado, Verônica Marques Zembrzuski, Mario Campos Junior, Clarissa Menezes Maya-Monteiro, Pedro Hernán Cabello, João Regis Ivar Carneiro, Patrícia Torres Bozza, Ana Carolina Proença da Fonseca

**Affiliations:** ^1^ Laboratory of Immunopharmacology, Oswaldo Cruz Institute, Oswaldo Cruz Foundation, Rio de Janeiro, Brazil; ^2^ Human Genetics Laboratory, Oswaldo Cruz Institute, Oswaldo Cruz Foundation, Rio de Janeiro, Brazil; ^3^ Clementino Fraga Filho University Hospital, Federal University of Rio de Janeiro, Rio de Janeiro, Brazil; ^4^ Birth Defects Epidemiology Laboratory, Oswaldo Cruz Institute, Oswaldo Cruz Foundation, Rio de Janeiro, Brazil; ^5^ Josué de Castro Nutrition Institute, Federal University of Rio de Janeiro, Rio de Janeiro, Brazil; ^6^ Genetics Laboratory, Grande Rio University/AFYA, Rio de Janeiro, Brazil; ^7^ Postgraduate Program in Translational Biomedicine, Grande Rio University/AFYA, Rio de Janeiro, Brazil

**Keywords:** genetic obesity, copy number variations, *SH2B1*, bariatric surgery, MLPA, CGH-array, severe obesity

## Abstract

**Introduction:**

Genetic obesity is considered a rare disease, affecting up to 10% of patients with severe early-onset obesity. Over the past years, significant advances have been made; however, the majority of patients are misdiagnosed with polygenic obesity. Thus, this study aimed to identify deleterious copy number variations (CNVs) linked to obesity and explore the clinical phenotypes.

**Method:**

The sample comprised 195 adults with severe obesity (BMI≥35kg/m^2^) who developed this phenotype during childhood or adolescence. We investigated the CNV using Multiplex Ligation-dependent Probe Amplification [MLPA] and real-time PCR. Chromosomal microarray analysis was used to assess the extent of genomic alterations.

**Results:**

One patient showed a ~206 kb deletion in the distal of the 16p11.2 region, encompassing twelve genes. The gene linked to the development of severe obesity was *SH2B1.* This alteration was found in a male patient with metabolic syndrome (MS), and hypertension. Two patients exhibited a large deletion in the proximal of the 16p11.2 region. One patient showed a ~534 kb deletion without twenty-nine genes. This female patient had hypertension and bronchitis. The other patient presented a ~598 kb deletion of the proximal 16p11.2 region, including thirty-two genes. This female patient exhibited MS, and moderate binge-eating disorder.

**Conclusion:**

Our study showed three genomic deletions at the 16p11.2 region in patients with severe obesity from Brazil. These results support the clinical utility of genetic testing to identify patients with the genetic form of obesity who may benefit from specific medical treatment, family genetic counseling, and targeted therapeutic intervention.

## Introduction

Obesity is defined as excessive fat accumulation, which may impair health (1). The common form of obesity has a multifactorial etiology; however, monogenic forms were identified in human (2). Genetic obesity is a rare disease, affecting up to 10% of patients with extreme and early-onset obesity (3). It occurs due to single-gene alterations or genomic copy number variants (CNVs), leading to both syndromic and non-syndromic forms of obesity. The main difference is the presence of development delay and intellectual deficit in syndromic obesity (4, 5). Several genes were identified as potential causes of non-syndromic monogenic obesity (NSMO), most of which are involved in hypothalamus development and/or the leptin-melanocortin pathway. These patients exhibit severe obesity with early onset and hyperphagia, caused by disruption of the satiety systems (2, 5, 6). In Brazil, previous results of our group identified 7 potential pathogenic variants in adult patients with severe obesity (5.7%), who developed this phenotype during childhood (7–11).

In recent years, new technological developments have allowed the characterization of new genetic forms of monogenic obesity due to CNVs. These genomic variations in several chromosomal regions were described in patients with genetic forms of severe obesity. Examples include deletions of chromosome band 6q16 including the *SIM1* gene, 11p13 which includes the *BDNF* gene, the recurrent 220-kb deletion of distal 16p11.2 including the *SH2B1* gene (OMIM #613444), and the recurrent 600-kb 16p11.2 proximal deletion (OMIM #611913, unknown gene) (4, 12–14). Molecular diagnosis is crucial to detect patients who might benefit from specific medical management, target therapeutic intervention, and genetic counseling to the patient and family. Despite this importance, the majority of individuals with genetic obesity are underdiagnosed (15).

To our knowledge, no reports of CNVs in Brazilian patients with NSMO were found in the literature. Thus, this study aimed to investigate potential CNVs linked to obesity and explore the clinical phenotype of the patients.

## Material and methods

### Patients

This cross-sectional observational study included 195 adult subjects with severe obesity and early onset from Rio de Janeiro, Brazil. Inclusion criteria were: severe obesity phenotype (BMI ≥ 35 kg/m^2^), developed during childhood (0-11 years, n=128 individuals) or adolescence (12-21 years, n=67 individuals), and also a candidate for bariatric surgery in our country. The period of obesity onset was self-reported. Exclusion criteria were: (1) pregnancy, (2) lactation, and (3) the presence of symptoms suggestive of syndromic obesity (cognitive delay, dysmorphic characteristics, and organ-specific developmental abnormalities). All patients were volunteers of a non-governmental organization, Rescue Group to Self-Esteem and Citizenship of the Obese (in Portuguese, “Grupo de Resgate à Autoestima e Cidadania do Obeso”). This study was approved by the Ethics Committee of the Oswaldo Cruz Foundation (CAAE:09225113.0.0000/Protocol N°: 346.634) and informed written consent was obtained from all participants before inclusion in this study.

Blood samples were collected after a 12-hour overnight fasting period. A metabolic workup was obtained, including a lipid profile, fasting glucose, glycated hemoglobin, reactive C PCR, blood pressure, and anthropometric parameters (weight, height, waist, hip, and neck circumference). We also measured serum concentration of leptin, resistin, monocyte chemoattractant protein-1/CCL2 (MCP1), and plasminogen activator inhibitor-1 (PAI-1) by Human Adipocyte Magnetic Bead (Millipore-Merck [cat# HADCYMAG-61 k]) on Bio-Plex 200 Multiplexing Analyzer System, according to the manufacturer’s protocol.

### Copy number variation analyses

Genomic DNA was isolated from peripheral blood using the QIAamp Blood Kit (Qiagen, Valencia, CA, USA). The DNA concentration was measured using the Nanodrop spectrophotometer (Thermo Fisher Scientific, Waltham, MA, United States).

CNV of the *BDNF* gene was investigated by real-time PCR assay using SYBR Green I chemistry and StepOne^®^ Plus Real-Time PCR System. *ALB* gene, located in 4q11, was used as a reference gene of invariable copy number variation to calculate the relative *BDNF* gene copy number. Two sets of primers were designed using Primer3Plus software, and are available upon request. All reactions were carried out in triplicate and a melting curve analysis was done to certify the specificity of each primer product.

CNV of the *LEPR*, *POMC*, *SIM1*, *LEP*, *MC4R*, *MC2R*, and *MC3R* genes and the 16p11.2 region were determined by multiplex ligation-dependent probe amplification (SALSA MLPA Kit P220-B3 Obesity; MRC Holland, Amsterdam, Netherlands). Labeled MLPA products were detected by ABI Prism 3130xl Genetic Analyzer (Applied Biosystems) with GeneScan500 LIZ size standard (Life Technologies). Data were analyzed with GeneMarker^®^ v.2.2.0 (Soft Genetics). The gene deletion or duplication was confirmed using quantitative real-time PCR (primers are available upon request). Then, chromosomal microarray analysis (CGH-array) was carried out to validate and identify the genomic breakpoints based on the human genome build GRCh38 (hg38).

American College of Medical Genetics and Genomics guidelines were used to classify the identified CNVs into 4 categories: pathogenic, likely pathogenic, variants of uncertain significance (VUS), and likely benign (16).

### Functional enrichment analysis of deleted genes in the CNV

The enrichment analysis of the list of genes affected by the deleted region in three obese patients was submitted to ToppGene (17). To facilitate a more comprehensive visualization of the results, a bar plot was constructed using the ggplot2 package (18) to display top 20 biological processes associated with these genes. The Venn diagram was constructed using the ggVennDiagram package. Additionally, the Venn diagram was generated using the ggVennDiagram package (19) in R version 4.4.1 (20) to illustrate the intersections of deleted genes across the three patients.

The targets of deleted microRNAs were found using miRWalk (21). The genes were considered targets of miRNAs, based on energy < -20 and the duplicated gene symbol was removed for each analysis (22). The Venn diagram was also constructed using the target intersection of both miRNA (https://bioinformatics.psb.ugent.be/webtools/Venn/). The enrichment analysis from genes target of miRNAs was released using web approach Metascape which aggregates 40 different databases to investigate function enrichment (23).

## Results

### Basic and genomic characteristics of the study population

This study included 195 unrelated Brazilian patients with severe obesity and early-onset. The basic clinical characteristics of the cohort are presented in **Table 1**.

**Table 1:** Basic characteristics of the study population.

Parameters	All		Childhood-Onset Obesity		Adolescence-onset Obesity		*P*
	n	Values	n	Values	n	Values	
**Age (years)**	195	36.0 (28.0; 43.0)	128	37.0 (28.2; 45.7)	67	34.0 (28.0; 40.0)	0.051
Gender (female/male)							
Female	195	152 (77.9)	128	99 (77.3)	67	53 (79.1)	0.465
Male		43 (22.1)		29 (22.7)		14 (20.9)	
Race/Skin color							
White	178	63 (35.4)	118	41 (34.7)	60	22 (36.7)	0.482
Brown		60 (33.7)		32 (27.1)		20 (33.3)	
Black		52 (29.2)		42 (35.6)		18 (30)	
Others		3 (1.7)		3 (2.6)		0 (0.0)	
Marital status							
Single	163	71 (43.5)	105	38 (36.2)	58	33 (56.9)	0.622
Married/cohabiting		75 (46.01)		51 (48.6)		24 (41.4)	
Separated/divorced		11 (6.7)		10 (9.5)		1 (1.7)	
Widower		6 (3.8)		6 (6.3)		0 (0.0)	
Smoking status							
Already smoked	180	15 (8.3)	120	10 (8.3)	60	5 (8.3)	**0.013**
Never smoked		165 (91.7)		110 (91.7)		55 (91.7)	
Physical activity practice							
Yes	182	46 (25.3)	122	36 (29.5)	60	10 (16.7)	0.061
No		136 (74.7)		86 (70.5)		50 (83.3)	
**Weight (kg)**	195	130.5 (112.9; 146.6)	128	131.2 (114.5; 149.3)	67	128.4 (111.7; 145.1)	0.655
**Height (m)**	195	1.64 (1.58; 1.70)	128	1.64 (1.58; 1.70)	67	1.63 (1.60; 1.70)	0.905
**BMI (kg/m^2^)**	195	47.5 (42.8; 54.4)	128	47.4 (43.2; 53.2)	67	47.5 (42.6; 54.9)	0.855
**Waist circumference (cm)**	194	137.0 (126.0; 147.1)	128	137.5 (126.1; 145.9)	66	136.0 (123.7; 147.9)	0.555
**Hip circumference (cm)**	194	142.0 (133.0; 152.0)	128	141.7 (133.2; 152.2)	66	142.5 (133.0; 152.2)	0.863
**WHR**	194	0.96 (0.91; 1.01)	128	0.97 (0.91; 1.01)	66	0.95 (0.89; 1.00)	0.126
**BAI**	190	49.5 (44.9; 55.1)	124	48.4 (44.7; 55.6)	66	50.6 (45.3; 54.0)	0.799
**Glucose (mg/dl)**	137	96.0 (89.5; 107.0)	85	97.0 (91.5; 105.5)	52	94.5 (89.0; 111.2)	0.511
**Total cholesterol (mg/dl)**	161	193.0 (168.0; 220.0)	102	193.0 (166.0; 224.0)	59	192.0 (170.0; 214.0)	0.629
**HDL cholesterol (mg/dl)**	161	46.0 (41.0; 52.0)	102	45.0 (40.0; 52.2)	59	48.0 (43.0; 52.0)	0.242
**LDL cholesterol (mg/dl)**	157	121.0 (97.5; 139.5)	99	123.0 (98.0; 143.0)	58	116.0 (96.7; 133.0)	0.246
**Triglycerides (mg/dl)**	161	124.0 (91.5; 166.5)	102	125.0 (86.7; 167.2)	59	122.0 (99.0; 160.0)	0.854
**Hemoglobin glycated (%)**	110	5.70 (5.10; 6.30)	75	5.70 (5.10; 6.40)	35	5.70 (5.00; 6.10)	0.279
**CRP (mg/dL)**	109	1.11 (0.60; 2.21)	78	1.31 (0.57; 2.42)	34	0.82 (0.61; 1.65)	0.204
**SBP (mm Hg)**	105	131.0 (119.5; 147.0)	67	131.0 (121.0; 148.0)	38	131.0 (114.0; 143.2)	0.437
**DBP (mm Hg)**	105	85.0 (76.5; 91.5)	67	86.0 (78.0; 91.0)	38	83.5 (74.7; 92.7)	0.447
**Leptin (pg/mL)**	91	2.645.4 (2,099.6; 3,388.5)	91	2.645.4 (2,099.6; 3,388.5)		na	
**MCP1 (pg/mL)**	91	254.4 (144.1; 384.2)	91	254.4 (144.1; 384.2)		na	
**PAI1 (pg/mL)**	91	26,257.8 (18.507.5; 31,688.34)	91	26,257.8 (18.507.5; 31,688.34)		na	
**Resistin (pg/mL)**	91	8,512.6 (5,874.7; 10,934.04)	91	8,512.6 (5,874.7; 10,934.04)		na	

*P*-value for differences between childhood- and adolescence-onset obesity. Bold font indicates statistical significance. Data are presented as median (interquartile) for continuous variables and number (percentage) for qualitative variables. BAI, body adiposity index; BMI, body mass index; CRP, C-reactive protein; DBP, diastolic blood pressure; HDL-cholesterol, high-density lipoprotein-cholesterol; LDL-cholesterol, low-density lipoprotein-cholesterol; MCP1, monocyte chemoattractant protein-1; na, not available; PAI-1, plasminogen activator inhibitor-1; SBP, systolic blood pressure; WHR, waist-hip ratio. Biochemical range reference: Glucose, 70 e 99 mg/dL; Total cholesterol, <199 mg/dL; HDL cholesterol, >40 mg/dL; LDL cholesterol, <100 mg/dL; Triglycerides, <150 mg/dL; Hemoglobin glycated, <5.7%; CRP, <0.3 mg/dL.

Copy number imbalances were found in three individuals (diagnostic yield of 1.5%). The genomic alterations were confirmed by real-time PCR and CGH-array. All three patients were found with pathogenic CNVs at the 16p11.2 locus, of which one was restricted to the distal region flanked by segmental duplications (SD) blocks breakpoint (BP)2 and BP3, and two to the proximal region flanked by SD blocks BP4 and BP5 (**Supplementary Figure S1**). All CNVs were in heterozygosity.

### Clinical features of the patients with CNVs

**Figure 1:**
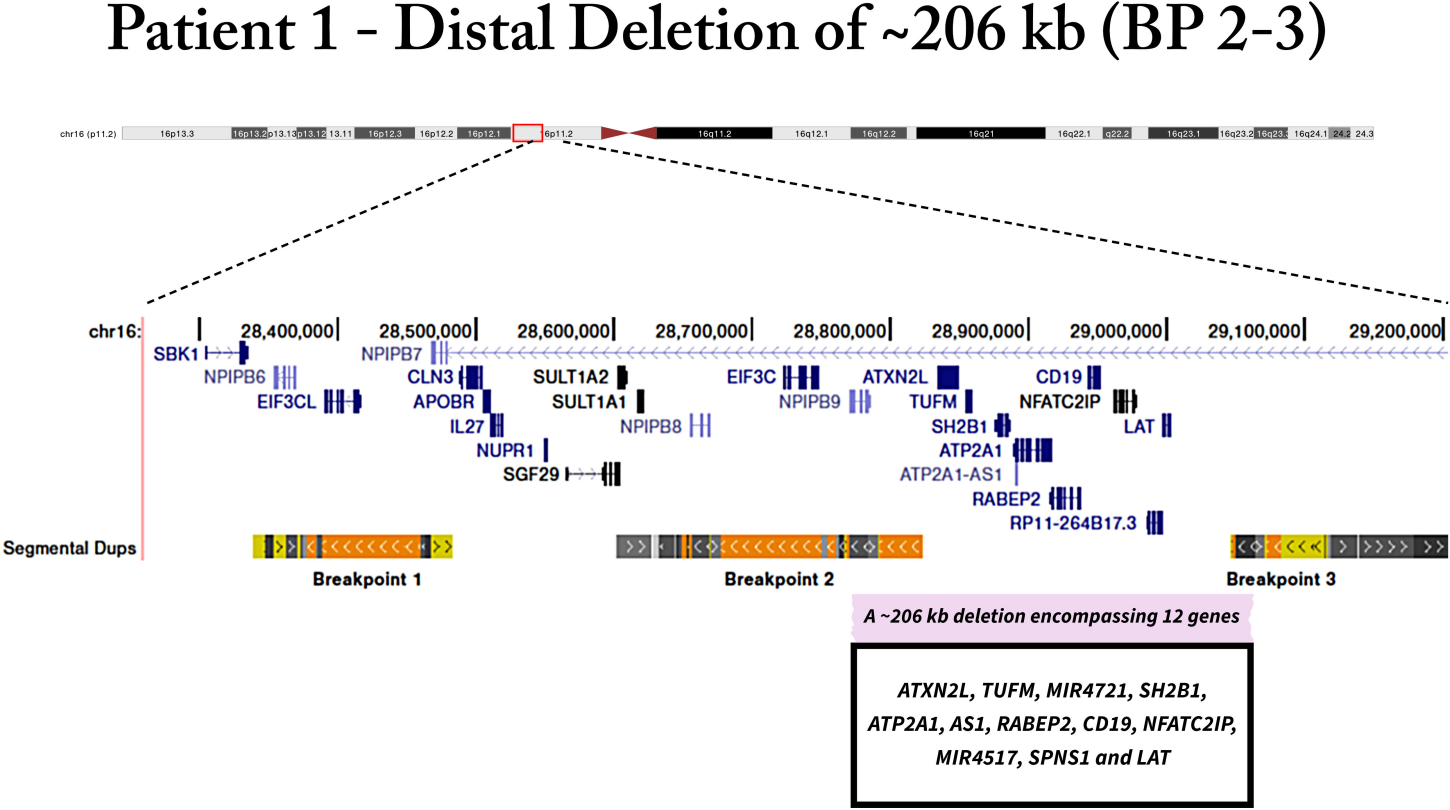
Genomic deletion of patient 1 in 16p11.2 region, which includes SH2B1 gene. The deletion encompasses twelve genes.

Patient 1 had deletion for three probes from 16p11.2 in MLPA, showing a complete deletion of the *SH2B1* gene. The deletion was shown to have a size of ~206 kb in the distal of the 16p11.2 region, encompassing twelve genes (*ATXN2L*, *TUFM*, *MIR4721*, *SH2B1*, *ATP2A1*, *AS1*, *RABEP2*, *CD19*, *NFATC2IP*, *MIR4517*, *SPNS1* and *LAT*) (**Figure 1**). The patient was a 19-year-old male individual, which reported severe early-onset obesity (childhood). His body weight was 127.5 kg for 1.62 m of height with a BMI of 48.6 kg/m^2^. The waist circumference was 138 cm; hip circumference was 134 cm; WHR, 1.03; neck circumference, was 44 cm. We measured biochemical parameters, in which fasting plasma glucose was 112 mg/dl, total cholesterol, 281 mg/dl; HDL-c, 53 mg/dl; LDL-c, 175 mg/dl; TG, 263 mg/dl; glycated hemoglobin, 5,2%; and reactive C protein, 0.76 mg/l. The patient had hypertension and metabolic syndrome.

**Figure 2:**
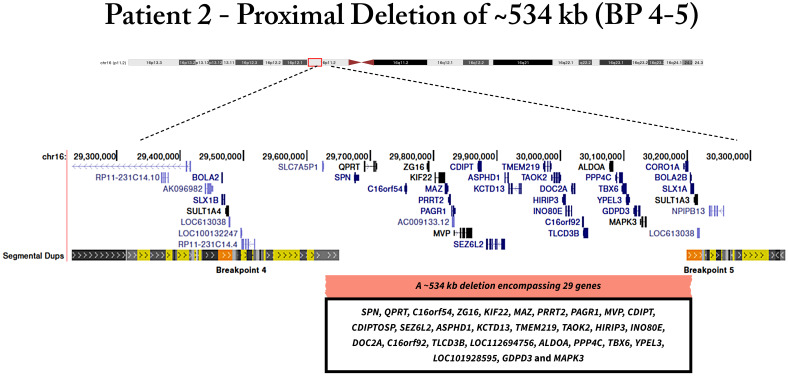
Genomic deletion of patient 2 located in proximal region of 16p11.2, encompassing twenty-nine genes.

Patients 2 and 3 presented a deletion in the *SEZ6L2* gene probe, localized in the 16p11.2 region. Further analyzes showed that patient 2 exhibited a ~534 kb deletion of proximal 16p11.2 locus, encompassing twenty-nine genes (*SPN, QPRT, C16orf54, ZG16, KIF22, MAZ, PRRT2, PAGR1, MVP, CDIPT, CDIPTOSP, SEZ6L2, ASPHD1, KCTD13, TMEM219, TAOK2, HIRIP3, INO80E, DOC2A, C16orf92, TLCD3B, LOC112694756, ALDOA, PPP4C, TBX6, YPEL3, LOC101928595, GDPD3, MAPK3)* (**Figure 2**). The deletion was detected in a 45-year-old female patient, who developed obesity during adolescence. Her body weight was 112.9 kg for 1.51 m with a BMI of 49.5 kg/m2. Her waist circumference was 144 cm; hip circumference was 147 cm; WHR, 0.98; neck circumference, was 47 cm. The blood pressure was 142/71 mmHg. Biochemical parameters were obtained, in which fasting plasma glucose was 86 mg/dl; total cholesterol, 235 mg/dl; HDL-c, 57 mg/dl; LDL- c, 151 mg/dl; TG, 134 mg/dl. The patient exhibited hypertension and bronchitis.

**Figure 3:**
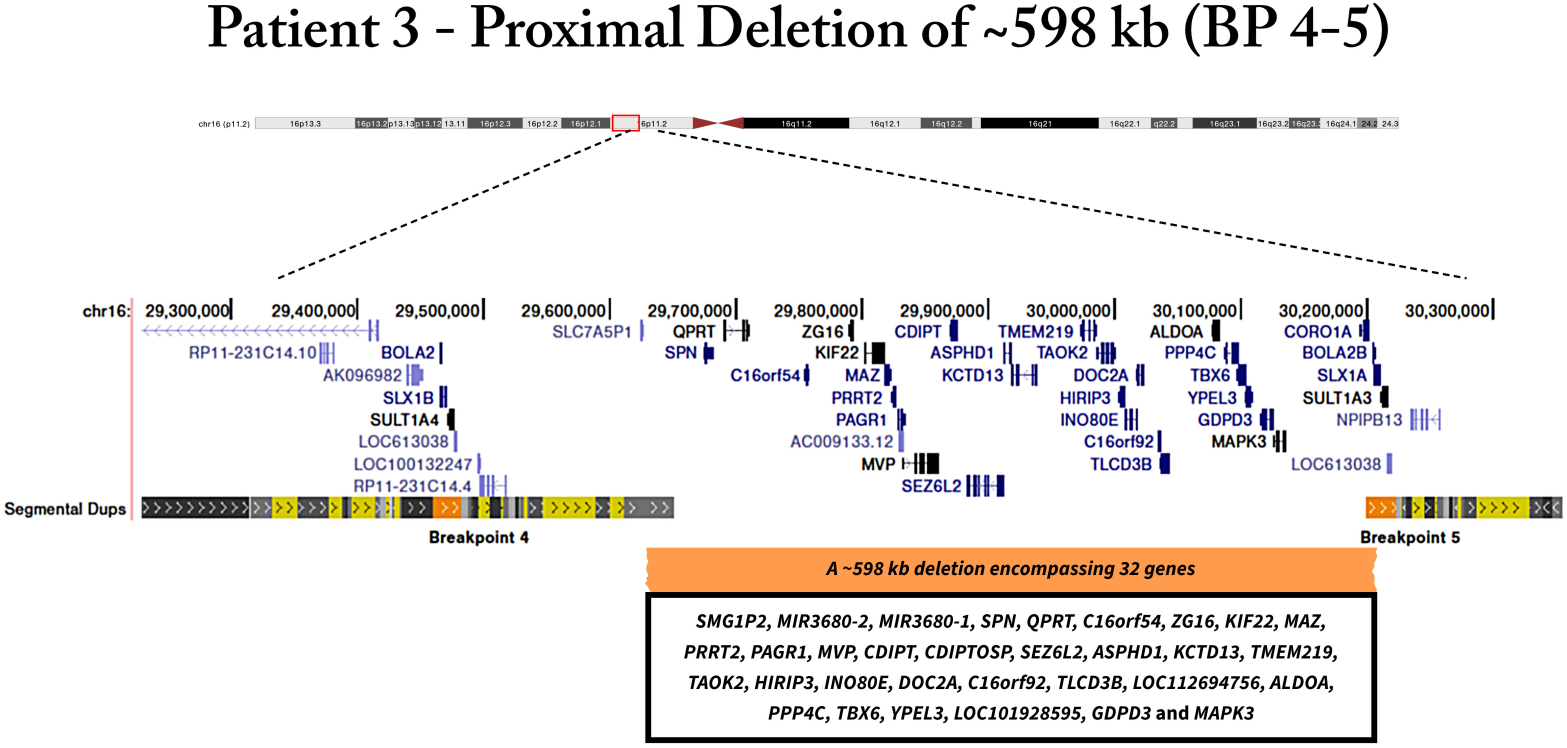
Genomic deletion of patient 3 located in 16p11.2 locus, encompassing thirty-two genes.

Patient 3 presented a ~598 kb deletion of proximal 16p11.2 region that includes thirty-two genes (*SMG1P2, MIR3680-2, MIR3680-1, SPN, QPRT, C16orf54, ZG16, KIF22, MAZ, PRRT2, PAGR1, MVP, CDIPT, CDIPTOSP, SEZ6L2, ASPHD1, KCTD13, TMEM219, TAOK2, HIRIP3, INO80E, DOC2A, C16orf92, TLCD3B, LOC112694756, ALDOA, PPP4C, TBX6, YPEL3, LOC101928595, GDPD3, MAPK3*) (**Figure 3**). The patient 3 was a 31-year-old female with severe early-onset obesity (during childhood). Her body weight was 96.3kg in 1.55 m with a BMI of 40.1 kg/m^2^. The waist circumference was 115 cm; hip circumference was 130 cm; WHR was 0.88, and neck circumference, was 39.5 cm. Regarding biochemical parameters, fasting glucose was 62 mg/dl; total cholesterol, 206 mg/dl; HDL-c, 43 mg/dl; LDL- c, 143 mg/dl; TG, 100 mg/dl. We also measured the concentration of serum hormones and cytokines, leptin, 2,656.19 pg/m; MCP1, 133.07 pg/ml; PAI-1, 37,486.56 pg/ml; and resistin, 9,411.69 pg/ml. She presented metabolic syndrome and moderate binge-eating disorder (BED), consuming a median of 3,578.42 kcal per day.

### Functional enrichment analysis of deleted genes in the patients

Firstly, each patient’s signatures of common and specific genes deleted in the 16p11.2 region were analyzed (**Supplementary Figure S2**). Patient 1 has twelve specific deleted genes, which were presented in the other patients. Among these deleted genes, they act in different pathways related to the concentration and transport of micro and macromolecules; proliferation and differentiation of specific immune cells; and cytoskeleton regulation (**Supplementary Figure S3**).

Patients 2 and 3 had the same twenty-nine deleted genes; however, patient 3 had three more deleted genes. These common genes act in different biological processes, among them the main pathways are related to the development, differentiation, and communication of the central nervous system. For example, these genes are involved in the development of the brain and head; axonogenesis; and the development and differentiation central nervous system (**Supplementary Figure S4**).

The patient 3 has three specific genes deleted in the proximal 16p11.2 region. Two genes encode microRNA (miRNA) (*MIR3680-1* and *MIR3680-2*) and one is a pseudogene (*SMG1P2*). The target genes of MIR3680-1 and MIR3680-2 were found using miRWalk. Our data showed that miRNA-3860-5p regulates 1330 genes, while miRNA-3860-5p modulates 2630 genes (**Supplementary Tables S1**, **S2**, respectively). The target genes of miRNA3860, 5p and 3p, were compared to identify common genes regulated by this miRNA, indicating that both modulate 354 genes (**Supplementary Figure S5**). These common genes are involved in different biological processes, including axonogenesis and the regulation of the development of neuron projection and the nervous system (**Supplementary Figure S6**).

## Discussion

In the present study, we aimed to characterize the CNVs in genes related to NSMO by MLPA in patients with severe obesity and candidates for bariatric surgery in Brazil. In our cohort of 195 patients, 1.53% (3 individuals) had pathogenic deletions at the 16p11.2 region. In the short arm of chromosome 16, there are several flanking segmental duplications with a high degree of sequence identity, which could result in a recurrent rearrangement commonly caused by non-allelic homologous recombination events during meiosis. In this context, these genomic deletions are frequently not inherited from either parent but arise *de novo* (24).

The 16p11.2 distal deletion was found in one patient. This deletion has a size of ~206 kb, encompassing twelve genes. This locus includes the *SH2B adaptor protein 1* (*SH2B1*) gene, linked to the development of obesity phenotype. Recently, Hanssen et al. (25) have investigated the clinical spectrum associated with 16p11.2 distal deletion in adults from the United Kingdom (UK) and Estonian biobanks. They reported that heterozygous carriers of this deletion have obesity with early onset, and accelerated metabolic disease, especially early and difficult-to-treat type 2 diabetes. This deletion was shown to account for 0.016% of the UK and 0.021% of Estonian biobanks (25).

We also found two patients with the proximal 16p11.2 deletion. This deletion represents the second most frequent genetic cause after *MC4R* point variants (26). Interestingly, a reciprocal duplication can influence BMI in a converse manner, resulting in underweight. It indicates that 16p11.2 CNVs have mirror etiologies, probably due to contrasting effects on energy homeostasis (27). In this line, an earlier study reported an abnormal satiety response in proximal 16p11.2 deletion carriers. However, the mechanism underlying this energy imbalance is still unknown (28). Controversially, murine models carrying paralogous 16p11.2 deletions and duplications showed an inverse phenotype found in humans, in which deletion and duplication mice resulted in under- and overweight, respectively (29, 30).

The functional enrichment analysis of deleted genes in our patients was performed to investigate the biological processes that are affected by the deletions. Interestingly, our data indicated that the deleted genes in the proximal 16p11.2 region are involved in brain development, as well as central nervous system development, differentiation, and communication. This result suggests that this deletion may impair the brain region’s development and/or function related to energy homeostasis, especially the hypothalamus development and/or the leptin-melanocortin pathway. It important to highlight that patient 2 and 3 have twenty-nine common genes deleted; however, patient 3 has more three specific genes deleted. Two of them are miRNAs that act mainly in axonogenesis and nervous system development. Previous studies showed that obesity is associated with miRNA deregulation (31). Further studies are necessary to elucidate the relation of miRNA deletion effects in the patients with NSMO.

A previous study observed that the proximal 16p11.2 deletion results in a highly penetrant form of obesity in adults; however, childhood presented a more variable phenotype. In addition, they also observed a low penetrance and variable phenotype in neurodevelopment disorders, including developmental delay and cognitive deficit. It is estimated that this deletion is found in 0.4% of patients with severe obesity – in line with our results (1%) (26).

Windholz et al. (32) investigated CNVs in classical obesity candidate genes in 194 children with severe early-onset obesity from Germany. By MLPA, they have analyzed *POMC*, *LEP*, *LEPR*, *SIM1*, *MC4R*, *MC3R* and *MC2R* genes. Similar to our results, they did not find CNVs in these candidate genes (32). In Brazil, D’Angelo et al. (4) performed a chromosomal microarray analysis on 279 patients with syndromic obesity. They identified a *de novo* recurrent 220-kb deletion of distal 16p11.2 in a 7-year female patient with overweight (BMI 20.5 kg/m^2^; 96.6th). This patient showed a deletion of 7 genes, including *SH2B1*. In addition, they found the recurrent 600-kb 16p11.2 proximal deletion in a 12-year male patient with obesity (BMI 30.4 kg/m^2^; 98.8th). Interestingly, a duplication of this same genomic rearrangement was observed in an 8-year-old female, which was reported to present obesity. This CNV was paternally inherited (4).

It is important to note that in this previous study, all the patients presented syndromic obesity, while our study included only individuals with non-syndromic obesity. It suggested that these rare forms of obesity may be associated with syndromic phenotypes or not. Thus, there is significant phenotypic variability among carriers of 16p11.2 deletions, and the etiology remains unclear. One possible explanation is other rare and common genetic variants contributing to clinical and neurobehavioral phenotypes. In addition, penetrance is age-dependent and each individual should be considered for your specific features (12).

## Conclusion

Understanding the rare genetic forms of obesity etiology has proven difficult. Unfortunately, the majority of patients with NSMO are underdiagnosed, and the genetic diagnosis is crucial to adequate clinical management, family genetic counseling, or even in some cases, targeted therapeutic intervention (33). Here, we diagnosed three patients with NSMO caused by deletions in the 16p11.2 locus at both proximal and distal intervals. From a perspective, these results allow us to implement a quick and efficient diagnostic method for patients with these rare genetic forms in the future.

## Data Availability

The original contributions presented in the study are included in the **Supplementary Datasheet 1**. Further inquiries can be directed to the corresponding author.
