# Supplementary figures and images for: Genomic deletions on 16p11.2 associated with severe obesity in Brazil
Source: Front Endocrinol (Lausanne). 2025 Jan 17;15:1495534. doi: 10.3389/fendo.2024.1495534 (PMC11781945; doi:10.3389/fendo.2024.1495534)

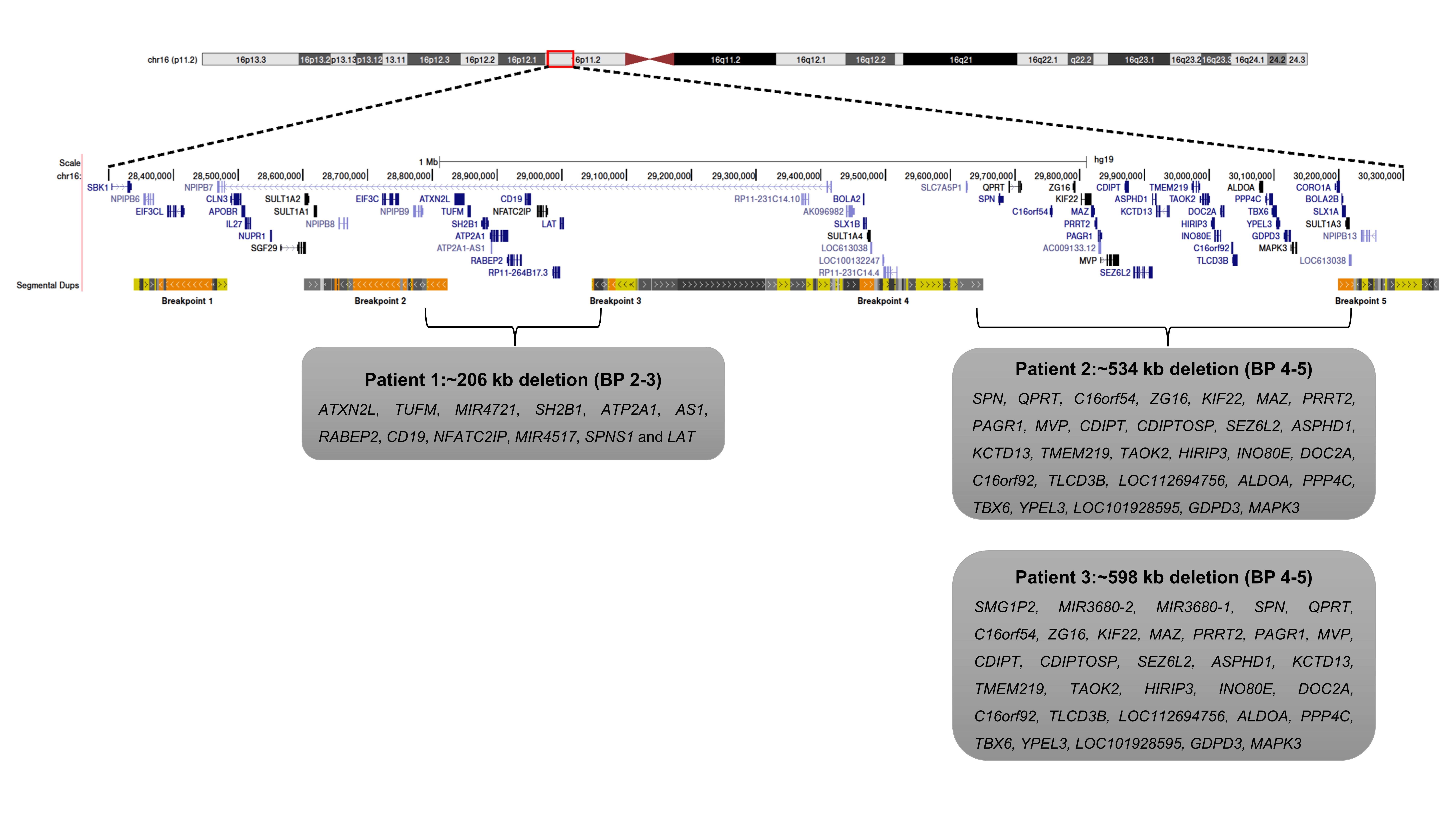

Supplement: Supplementary file 1 [file Image1.jpg]

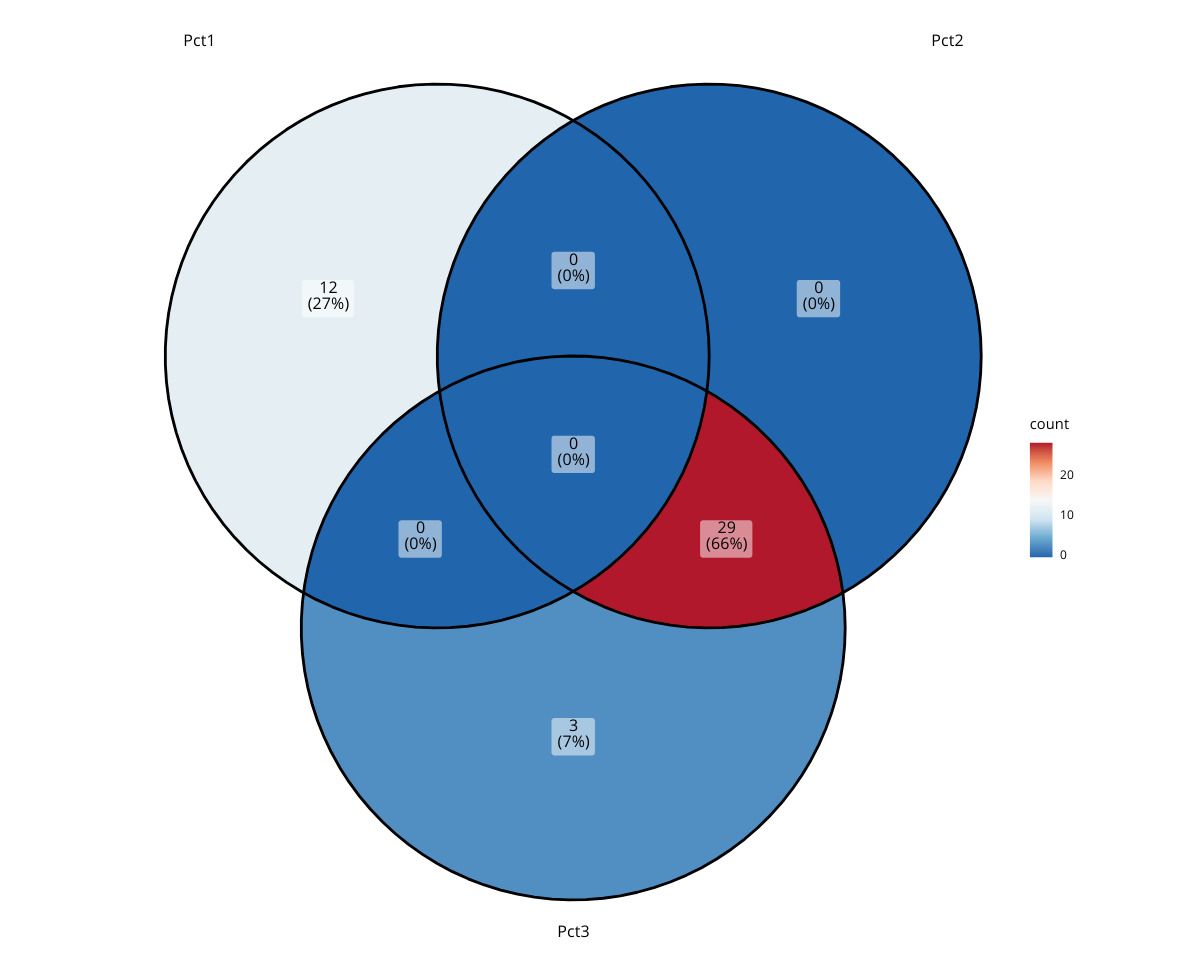

Supplement: Supplementary file 2 [file Image2.jpg]

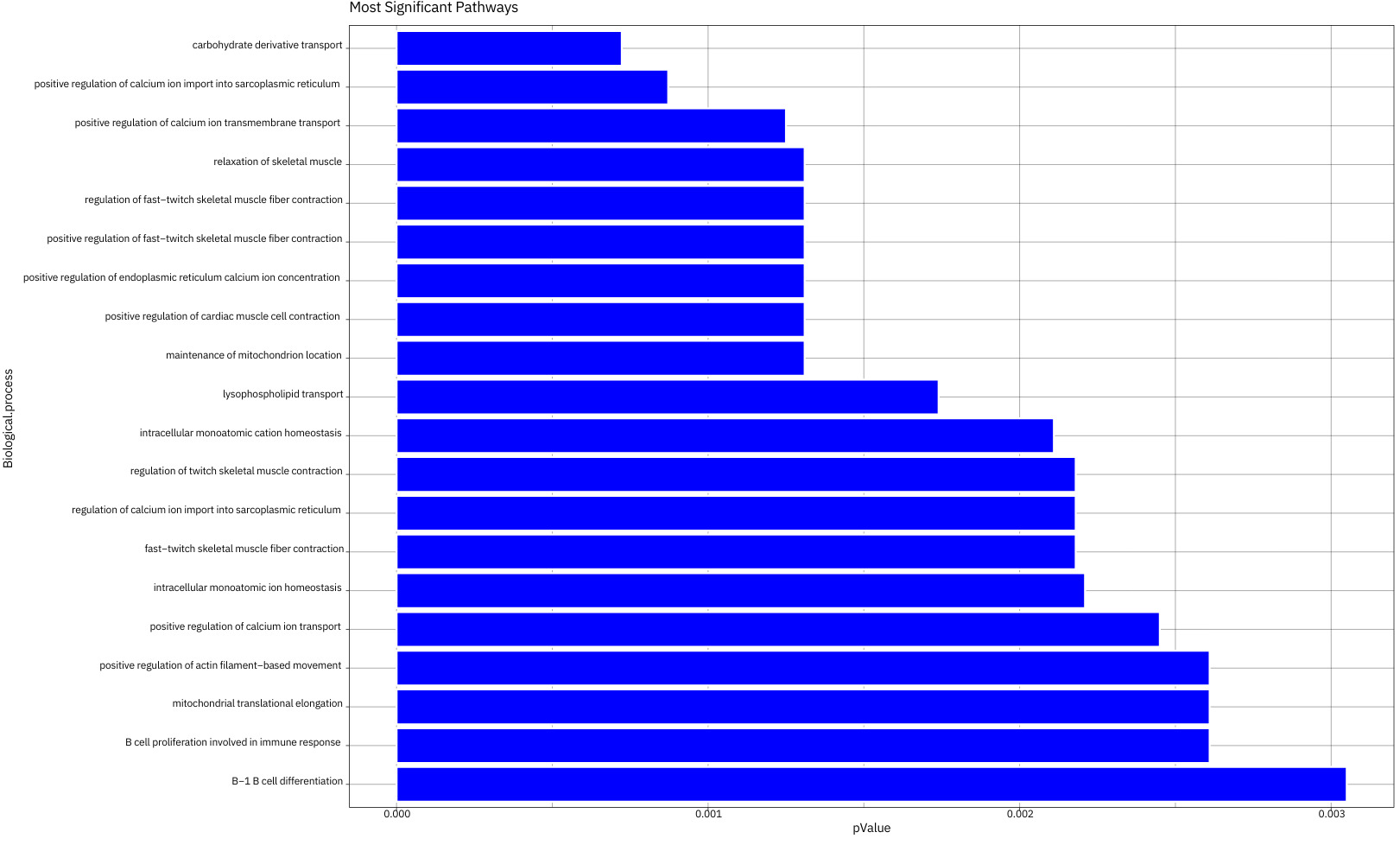

Supplement: Supplementary file 3 [file Image3.jpg]

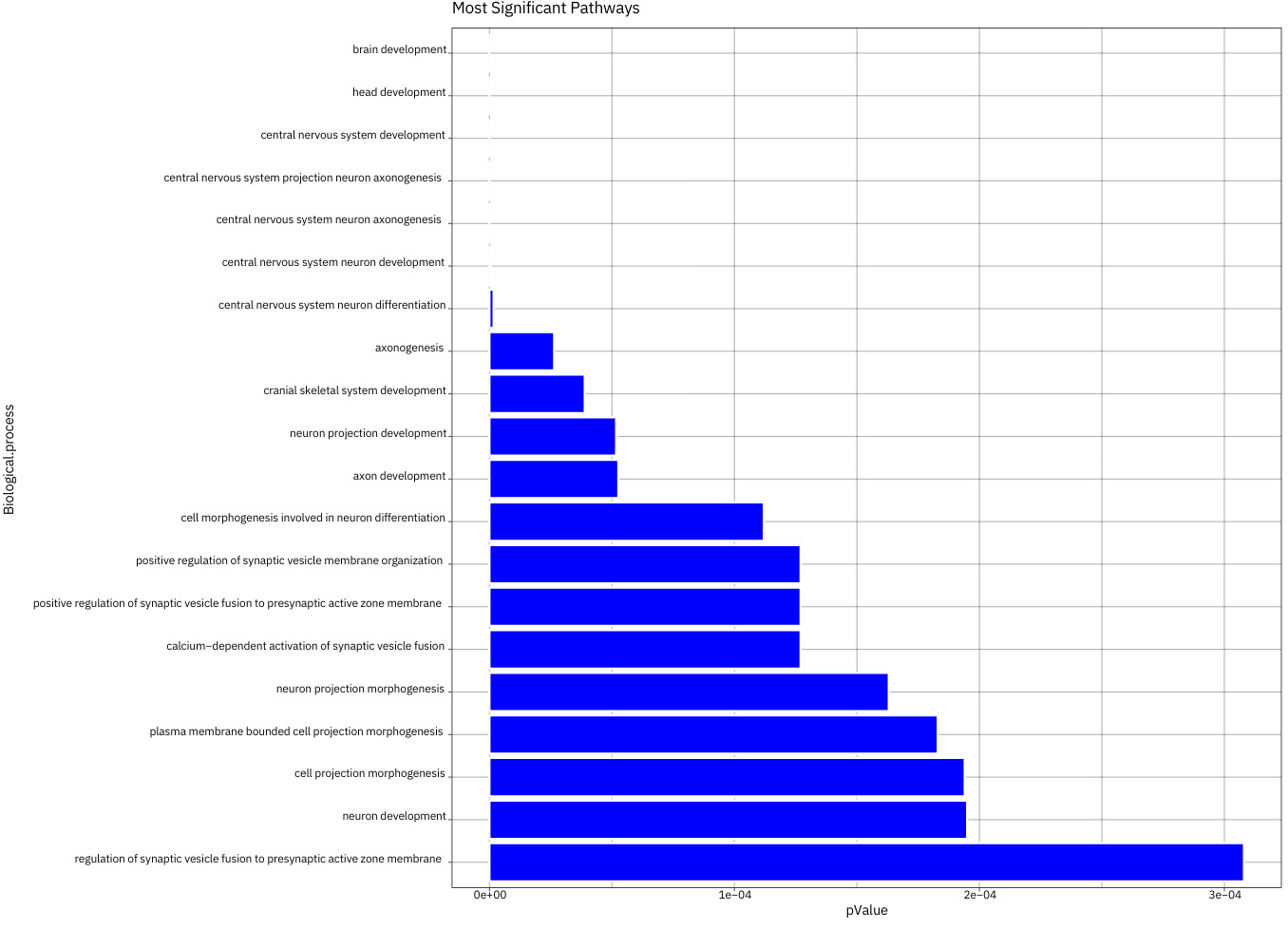

Supplement: Supplementary file 4 [file Image4.jpg]

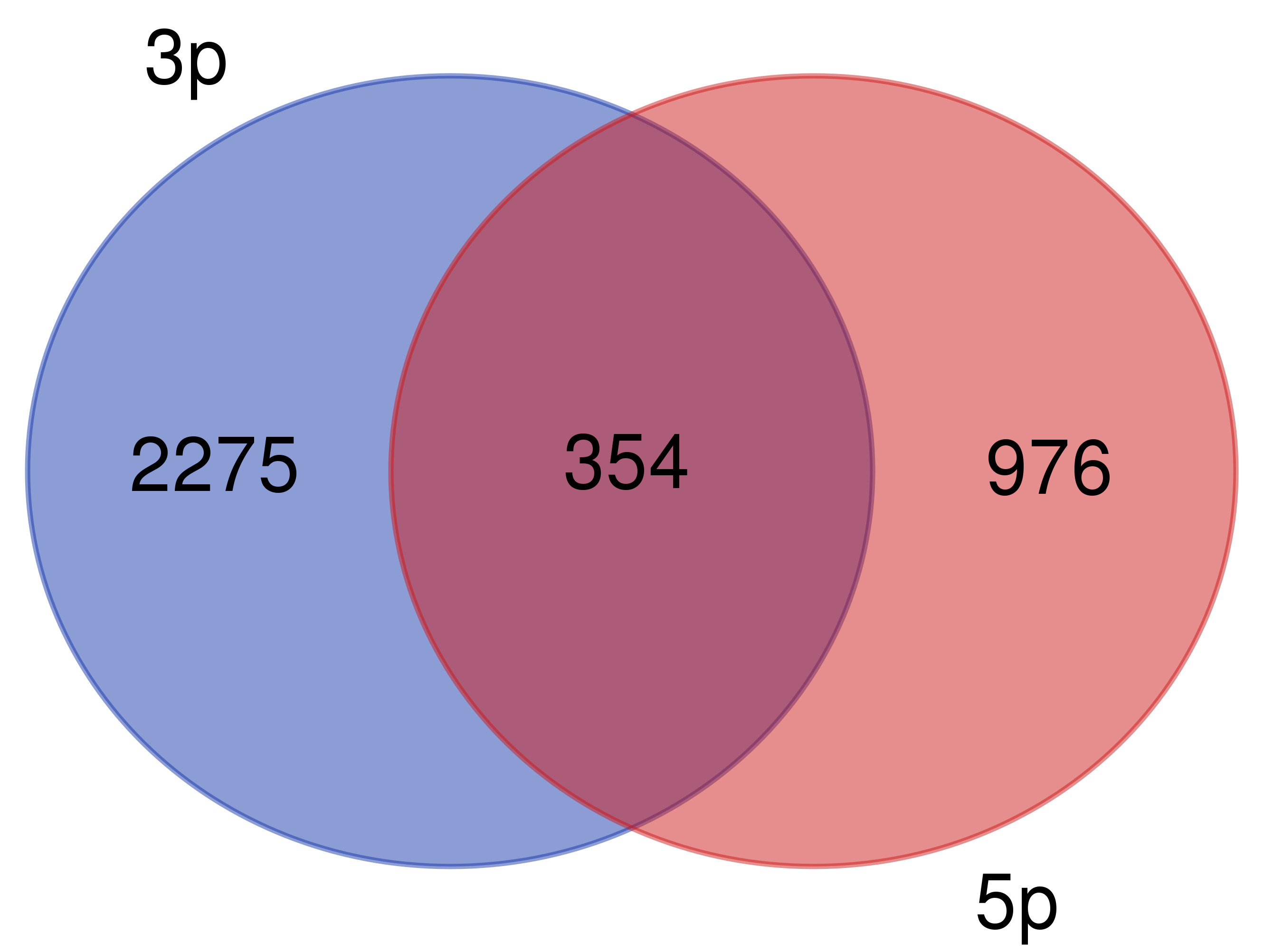

Supplement: Supplementary file 5 [file Image5.jpg]

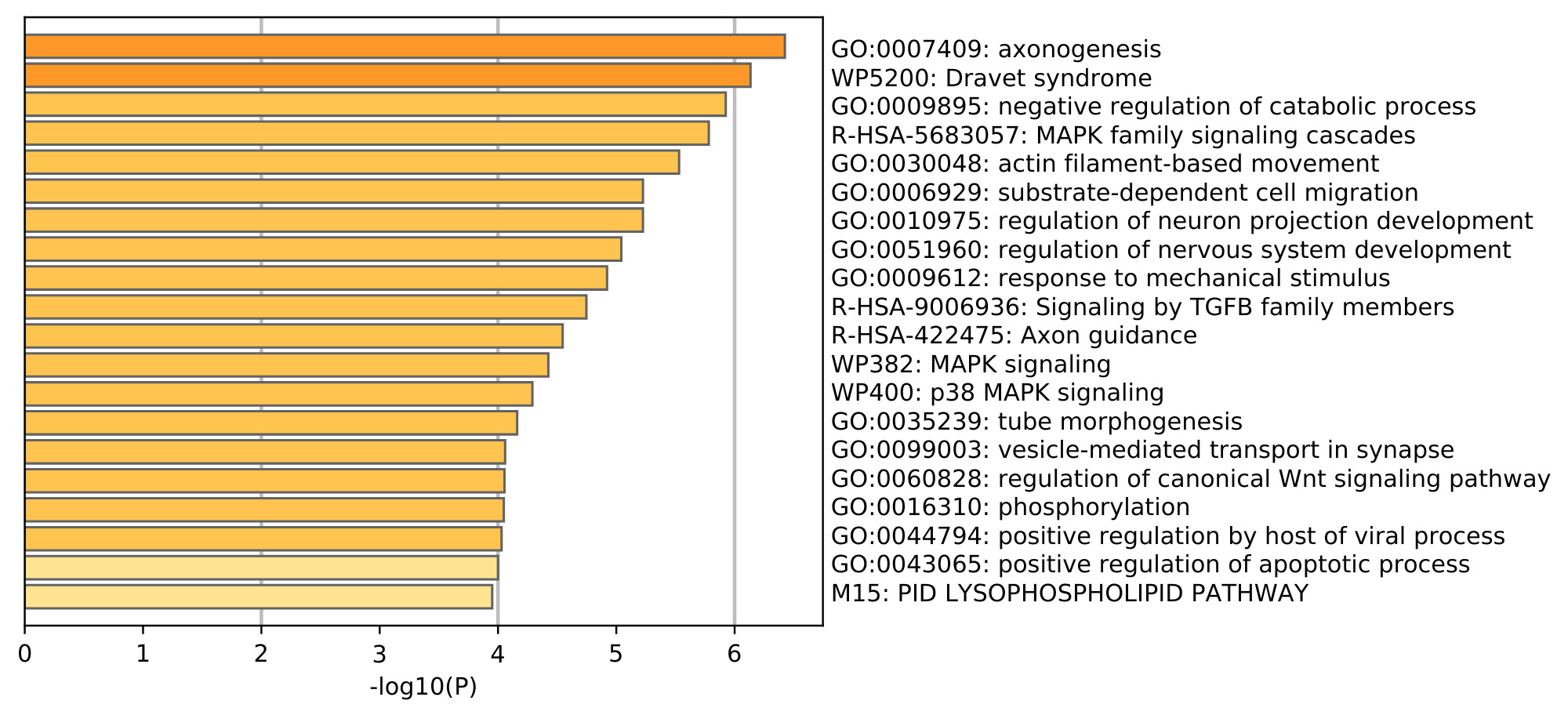

Supplement: Supplementary file 6 [file Image6.jpg]
